# Five Constituents Contributed to the Psoraleae Fructus-Induced Hepatotoxicity via Mitochondrial Dysfunction and Apoptosis

**DOI:** 10.3389/fphar.2021.682823

**Published:** 2021-12-07

**Authors:** Zhaojuan Guo, Pin Li, Chunguo Wang, Qianjun Kang, Can Tu, Bingqian Jiang, Jingxuan Zhang, Weiling Wang, Ting Wang

**Affiliations:** ^1^ Beijing Research Institute of Chinese Medicine, Beijing University of Chinese Medicine, Beijing, China; ^2^ NMPA Key Laboratory for Research and Evaluation of Traditional Chinese Medicine, Beijing University of Chinese Medicine, Beijing, China

**Keywords:** Psoraleae Fructus, mitochondrial membrane potential, reactive oxygen species, apoptosis, hepatotoxic ingredients

## Abstract

**Backgrounds:** Psoraleae Fructus (PF)-induced hepatotoxicity has been reported in clinical and animal experiments. However, the hepatotoxic constituents and mechanisms underlying PF-induced toxicity have remained unclear. Therefore, this study explored the potentially toxic PF components and revealed their relative mechanisms.

**Methods:** The hepatotoxicity of PF water (PFW) and ethanol (PFE) extracts was compared using Kunming mice. The different compositions between PFW and PFE, which were considered toxic compositions, were identified using the UHPLC-Q-Exactive MS method. Then, L02 and HepG2 cell lines were used to evaluate the toxicity of these compositions. Cell viability and apoptosis were determined through the Cell Counting Kit-8 (CCK-8) assay and flow cytometry, respectively. An automatic biochemical analyzer detected the aspartate aminotransferase (AST), alanine aminotransferase (ALT), and alkaline phosphatase (ALP). Lastly, we used high-content screening (HCS) to determine the levels of reactive oxygen species (ROS), lipid, and mitochondrial membrane potential (MMP).

**Results:** The ethanol extraction process aggravated the hepatotoxicity of PF, causing more severe injuries. The content of psoralen, isopsoralen, bavachin, psoralidin, bavachinin, neobavaisoflavone, and bakuchiol was higher in the PFE than PFW. Bavachin, psoralidin, bavachinin, neobavaisoflavone, and bakuchiol induced cell apoptosis and the AST, ALT, and ALP leakages. Furthermore, these five constituents increased intracellular lipid accumulation and ROS levels but decreased the MMP level.

**Conclusion:** The ethanol extraction process could induce severe PF hepatotoxicity. Bavachin, psoralidin, bavachinin, neobavaisoflavone, and bakuchiol are the main hepatotoxic ingredients. This mechanism could be associated with oxidative stress and mitochondrial damage-mediated apoptosis. Taken together, this study provides a basis for the clinical application of PF that formulates and improves its herbal standards.

## Introduction

Herb-induced liver injury (HILI) is severe liver damage associated with the use of Chinese medicine (CM), herbal medicine (HM), or their related products (Wang JB. et al., 2018). With the widespread successful application of Traditional Chinese medicine (TCM) and the continuous improvement in monitoring and reducing adverse drug reactions, HILI has been reported to be on the rise (Chalasani et al., 2014). Psoraleae Fructus (PF) is the dried matured fruit of *Psoralea corylifolia* L. (Committee, 2015). It is widely used to treat yang deficiency of the spleen and kidney in adult and pediatric patients in China, Korea, and Japan. Furthermore, modern pharmacological studies have confirmed that this seed exhibits potent medicinal activities like estrogenic (Liu et al., 2020), antitumor (Jin et al., 2020), anti-oxidant (Limper et al., 2013), antimicrobial (Huang et al., 2014), anti-inflammatory (Szliszka et al., 2011), antidepressant (Mao et al., 2014), and osteoblastic activities (Huang et al., 2019). However, recent clinical reports have indicated that treatment associated with PF has an increased risk of liver injury (Nam et al., 2005; Teschke and Bahre, 2009; Smith and MacDonald, 2014; Li and Huang, 2016; Li and Cheng, 2018; Zhang and Huang, 2018). For instance, different studies have demonstrated that among 595 HILI patients, those who received PF-related prescriptions accounted for 40 cases (Zhu et al., 2016). Elsewhere, by utilizing the Chinese patent medicine containing PF (provided by the National Healthcare Directory), 42 patients suffered from liver injury (Wang Y. et al., 2018), and this problem has received much attention. The use of 70% and 80% ethanol extract of PF markedly increased the activity of alkaline phosphatase (ALP) and total bilirubin (TBIL), leading to hepatocyte hypertrophy, steatosis, and a decrease in the total hepatic sinusoid surface area (Wang et al., 2012; Wang et al., 2019 Y.). Moreover, the aqueous extracts of PF have also been reported to cause hepatotoxicity (Zhou et al., 2013; Bi et al., 2015; Maimaiti et al., 2017). Our previous long-term toxicity studies showed that mouse hepatotoxicity triggered by PF ethanol (PFE) extracts is more severe than PF water (PFW) extracts. Notably, the PFE-treated mouse liver showed moderate hepatocyte hypertrophy and steatosis in the central zone of the hepatic lobules (Guo et al., 2020). Therefore, PF is a potential hepatotoxin whose hepatotoxic components and underlying mechanisms remain obscure.

Previous PF-induced hepatotoxicity studies have primarily focused on the evaluation of the toxicity and mechanism of single compounds in PF, including bakuchicin (Kim et al., 2016), psoralen (Yu et al., 2020), isopsoralen (Yu et al., 2019), bakuchiol (Li ZJ. et al., 2017), and bavachinin (Wang S. et al., 2018). Nevertheless, it is well known that TCM has multi-component, multi-pathway, and multi-target characteristics. Hence, a single component cannot represent the hepatotoxicity of the whole herb. Thus, there is a need to develop a systematic method that discovers the toxic constituent of PF-induced liver injury.

The hallmark of drug-induced hepatotoxicity (DIH) is either the death of hepatocytes or sometimes cholangiocytes and endothelial cells (Shehu et al., 2017). Oxidative stress, generation of reactive metabolites, and mitochondrial dysfunction are common pathophysiological mechanisms of DIH (Liu et al., 2015). In particular, oxidative stress is the increased production of reactive oxygen species (ROS) that overwhelms the liver. It could damage the structural and functional integrity of cells in compound-dependent mechanisms underlying drug-induced liver injury (DILI) (Zhu et al., 2012; Singh et al., 2015). Mitochondria are some of the organelles implicated as the primary source of the generated ROS. However, excessive ROS production results from mitochondrial membrane peroxidation. Then, the mitochondrial membrane potential (MMP) collapses, ATP synthesis is blocked, and the intrinsic (mitochondrial) apoptosis pathways are activated and finally lead to cell death by apoptosis (Takemoto et al., 2014; Zorov et al., 2014; Granger and Kvietys, 2015; Iorga et al., 2017). Research has also reported that bavachin induced apoptosis in HepG2 cells, and ROS is an initial signal leading to the endoplasmic reticulum (ER) stress and mitochondrial dysfunction through the p38/JNK signaling pathway (Wang S. et al., 2018; Yang et al., 2018). In a nutshell, the above findings have suggested that PF could induce hepatotoxicity by accumulating ROS. Therefore, this study evaluates the hepatotoxicity of PF and explores the possible toxic mechanisms *in vitro*, including cell apoptosis, ROS, and MMP.

## Materials and Methods

### Reagents and Chemicals

The PF samples were purchased from Tongrentang Chinese Pharmaceutical Co. Ltd. (Beijing, China). All plant materials were identified by Prof. Xiangri Li, a Botanist Professor of the Beijing University of Chinese Medicine, Beijing, China.

Psoralen, isopsoralen, bavachin, psoralidin, bavachinin, neobavaisoflavone, and bakuchiol standards (˃98% purity) were procured from BioRuler (Danbury, CT, USA). Cell Counting Kit-8 (CCK-8) was bought from DOJINDO LABORATORIES (Kyushu, Japan). Dimethyl sulfoxide (DMSO) was obtained from Sigma-Aldrich (St. Louis, MO, USA). Annexin V-FITC/PI apoptosis detection kit was acquired from BD (Franklin Lakes, NJ, USA). Then, Hoechst 33342, eBioscience™ JC-1 Mitochondrial Membrane Potential Dye, Cell ROX Deep Red, and HCS LipidTOX™ Green Phospholipidosis Detection Reagent were obtained from Thermo Fisher Scientific (Waltham, MA, USA). Lastly, formic acid, methanol, and acetonitrile (Fisher, Fair Lawn, NJ, USA) were of high-performance liquid chromatography (HPLC) grade.

### Sample Collection and Preparation

The PF raw samples were processed according to the methods described in the 2015 edition of the National Pharmacopoeia Committee (General Rule 0213) to obtain their salt form. Water and ethanol extracts of PF were prepared through twice extraction for 1.5 h each and rinsed eight times using water or 70% ethanol by PF weight. The extracts were mixed, filtered, and concentrated under reduced pressure at 60°C using a vacuum drying oven. The yield ratios were 25.07% (w/w, PFW) and 65.40% (w/w, PFE), and the resulting samples were stored at 4°C awaiting further analyses.

### Animals

Thirty Kunming mice (7 weeks old) with body weights of 22–25 g were purchased from Beijing Vital River Laboratory Animal Technology Co., Ltd. [Permission No. SCXK (Beijing, China) 2016-0011]. They were kept in an environmentally controlled breeding room for 5 days at 22°C–25°C. The mice were housed with free access to laboratory food and water. After acclimatization, these mice were randomly divided into control, PFW, and PFE groups (n = 10). To dissolve the drugs, 0.5% CMC-Na was used. Mice in the treated groups were administered PFW or PFE at doses of 5.14 g/kg/day (dosage of PF) for 28 days; the control group was administered with the 0.5% CMC-Na. Then, the mice were sacrificed, and their livers were reserved for H&E staining. According to the *Guide for the Care and Use of Laboratory Animals*, all animal experiments were carried out and approved by the Ethical Committee on Animal Research at the Beijing University of Chinese Medicine.

### Transferase dUTP Nick-End Labeling (TUNEL) Assay

The sections of mouse liver tissues were stained and used to detect apoptosis using a commercial TUNEL Apoptosis Assay Kit (Meilun Bio, Dalian, China). The apoptosis ratio was analyzed under a fluorescence microscope by counting TUNEL-positive cells in six randomly selected areas. The apoptosis rate was calculated using the following formula:

Apoptosis rate(100%)=(number of TUNEL positive cells/number of total cells)×100%

### Analysis of Psoraleae Fructus Water and Psoraleae Fructus Ethanol Using the UHPLC-Q-Exactive MS Method

Methanol of 50-ml volume was added to moderate PFW and PFE powder equivalent to 1 g of PF and ultrasonically treated for 30 min using an ultrasonic cleaning instrument (KQ-500DB CNC; Kunshan Ultrasonic Instrument Co., Ltd., Kunshan, Jiangsu, China). The volume was then fixed to 50 ml by supplementing the lost volume with methanol. Notably, the extracts were filtered through 0.22-μm microporous membrane filters. Seven standards, including psoralen, isopsoralen, bavachin, psoralidin, bavachinin, neobavaisoflavone, and bakuchiol, were dissolved in methanol (0.10 mg/ml).

The chemical compounds of PFW and PFE were determined through the UHPLC-Q-Exactive MS method. Ultra-performance liquid chromatography (UPLC) analysis was performed on a Thermo Scientific Dionex UltiMate 3000 UHPLC system (Santa Clara, CA, USA) equipped with an ACQITY UPLC T3 column (2.1 mm × 100 mm, 1.8 μm; Waters, Milford, MA, USA). Subsequently, chemical compounds were kept at 30°C and a flow rate of 0.30 ml/min. The mobile phase consisted of acetonitrile (A) and 0.1% formic acid (B). The gradient elution of B was performed as follows: 95% B (0–3 min), 95%–25% B (3–45 min), 25%–95% B (45–45.1 min), and 95% B (45.1–50 min).

The UHPLC-Q-Exactive MS (Thermo Scientific, Santa Clara, CA, USA) technique was used to analyze and identify PFW and PFE chemical compounds. When these samples were analyzed in negative ion detection mode, heated electrospray ionization (HESI) was the ion source. The temperature was set at 350°C; the spray and capillary voltages were set to 3.0 kV and 35.0 V, respectively; and the tube lens voltage was 110 V. High-purity nitrogen (>99.99%) was used as the sheath (30 arb) and auxiliary (10 arb) gas. Subsequently, the samples were analyzed in positive ion detection mode. The ion source temperature was 350°C, and the spray and capillary voltages were set to 3.0 kV and 35.0 V, respectively. The tube lens voltage was 110 V. High-purity nitrogen (>99.99%) was used as the sheath (40 arb) and auxiliary (20 arb) gas.

### Cell Culture

L02 and HepG2 cells were purchased from the China Infrastructure of Cell Line Resources (Beijing, China). They were cultured in Dulbecco’s modified Eagle’s medium (DMEM) high glucose medium (Gibco, Grand Island, NY, USA) supplemented with 10% (v/v) fetal bovine serum (FBS) (Gibco, Melbourne, VIC, Australia) and 1% U/ml of antibiotics (penicillin and streptomycin) at 37°C in an atmosphere containing 5% CO_2_.

### Cell Viability Assay

The viability of L02 and HepG2 cells was determined using the CCK-8 assay. L02 and HepG2 cells (1 × 10^4^ cells/well) were seeded into 96-well plates for 12 h until they visibly reached confluence. Then, the adherent cells were treated with varying concentrations of psoralen (120.86, 161.34, 215.12, 286.83, 382.44, 509.92, 679.90, and 906.53 μmol/L), isopsoralen (161.15, 214.87, 286.64, 382.47, 509.88, 679.85, 906.53, and 1,208.64 μmol/L), bavachin (16.40, 21.95, 29.23, 38.97, 52.04, 69.37, 92.49, and 123.32 μmol/L), psoralidin (8.92, 11.89, 15.82, 21.17, 28.19, 37.58, 50.19, and 66.90 μmol/L), bavachinin (11.82, 15.72, 21.04, 28.01, 37.35, 49.88, 66.49, and 88.65 μmol/L), neobavaisoflavone (5.21, 9.30, 16.50, 29.41, 52.36, 93.07, 165.53, and 294.46 μmol/L), and bakuchiol (11.70, 15.60, 20.75, 27.77, 36.97, 49.30, 65.84, and 87.76 μmol/L). Consequently, these cells were incubated for 24 h, and 100 μl of 10% (v/v) CCK-8 medium solution was added to each well for 2 h. The absorbance (optical density (OD)) of the culture medium was detected at 450 nm using a Multiskan GO microplate reader (Thermo Fisher Scientific). Each experiment was repeated thrice, and each treatment was probed in at least six wells. The following equation was used to calculate the viability of the cells (%):

$$\mathrm{Cell Viability}\;\left(\text{\%}\right)=\frac{OD_{\mathrm{compound}}-OD_{\mathrm{blank}}}{OD_{\mathrm{control}}-OD_{\mathrm{blank}}}\text{×}\mathrm{100\%}$$

### Flow Cytometric Analysis

First, L02 and HepG2 cells were cultured in 6-well plates at a density of 5 × 10^5^ cells/well. Then, they were treated with bavachin (17, 34, and 68 μmol/L), psoralidin (11.5, 23, and 46 μmol/L), bavachinin (15, 30, and 60 μmol/L), neobavaisoflavone (23.5, 47, and 94 μmol/L), and bakuchiol (13, 26, and 52 μmol/L) for 48 h, harvested, and washed with PBS. The apoptosis rate was measured using Pharmingen™ FITC Annexin V Apoptosis Detection Kit I (BD, 556,547, USA) following the instructions from the manufacturer. With the use of a FACSCalibur cytometer (Becton Dickinson, San Jose, CA, USA), 1 × 10^4^ cells were acquired and analyzed. Notably, the Annexin V-PI-, Annexin V-PI+, Annexin V + PI-, and Annexin V + PI + cells represented viable cells, necrotic cells, early apoptotic cells, and late apoptotic cells, respectively.

### Activity Assessment of Aspartate Aminotransferase, Alanine Aminotransferase, and Alkaline Phosphatase in Cell Culture Medium

L02 and HepG2 cells were cultured in 6-well plates at a density of 2 × 10^5^ cells/well. Subsequently, they were incubated with bavachin, psoralidin, bavachinin, neobavaisoflavone, and bakuchiol for 24 h, and the cell supernatant was collected. Lastly, aspartate aminotransferase (AST), alanine aminotransferase (ALT), and ALP were detected using a CX4 Pro automatic biochemical analyzer (Beckman, Brea, CA, USA).

### Evaluation of the Liver Injury Induced by Toxic Compounds of Psoraleae Fructus

Here, L02 and HepG2 cells were plated at a density of 2 × 10^5^ cells/well in 96-well plates. Then, they were treated with bavachin (17, 34, and 68 μmol/L), psoralidin (11.5 23, and 46 μmol/L), bavachinin (15, 30, and 60 μmol/L), neobavaisoflavone (23.5, 47, and 94 μmol/L), and bakuchiol (13, 26, and 52 μmol/L) for 24 h. Then Hoechst 33342, eBioscience™ JC-1 Mitochondrial Membrane Potential Dye, CellROX Deep Red, and HCS LipidTOX™ Green Phospholipidosis Detection Reagent kits were used to characterize their cell counts, nuclear area, MMP, ROS, and intracellular lipid. Consequently, multi-parameter cytotoxicity was analyzed through high-content screening (HCS) analysis (IN Cell Analyzer 2500 HS; Cytiva, Marlborough, MA, USA). Then, a 20× objective was used to collect all images. Three independent wells were examined for each treatment, and 16 fields per well were captured during the analysis. Lastly, HCS analysis was performed using Automated Image and Cell Analysis software (IN Cell Analyzer 2000; USA).

### Statistical Analysis

All statistical data analyses were performed using GraphPad Prism version 8.0 (GraphPad Prism Software, 2012; La Jolla, CA, USA). One-way ANOVA was used to assess the significant differences between means. Statistical significance was indicated as *p* < 0.05 or *p* < 0.01. Data were presented as mean ± SD.

## Results

### Evaluation of the Liver Injury Induced by Psoraleae Fructus

**FIGURE 1:**
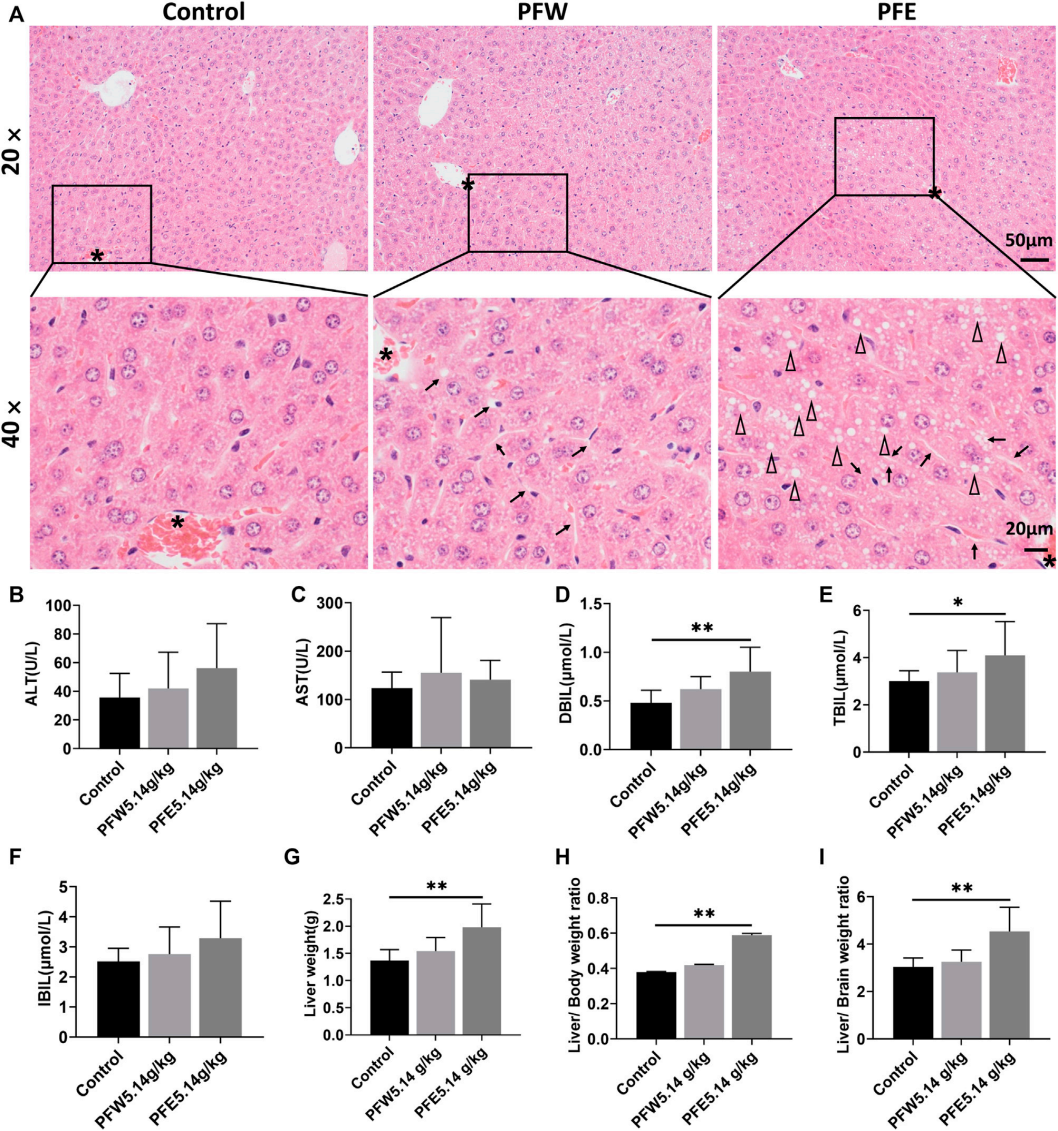
PFW- and PFE-induced liver injury. **(A)** Representative histopathological microphotographs of mouse liver illustrating the control, PFW, and PFE groups at ×20 (top line) and ×40 (bottom line) magnification. The asterisk indicates the central vein. Arrows indicate hepatocyte hypertrophy. Triangle indicates vacuolation. **(B)** ALT. **(C)** AST. **(D)** DBIL. **(E)** TBIL. **(F)** IBIL. **(G)** Liver weight. **(H)** Liver/body weight ratio. **(I)** Liver/brain weight ratio. Values are represented as mean ± SD, *n* = 10. ^*^ *p* < 0.05 and ^**^ *p* < 0.01 versus the control group.

**FIGURE 2:**
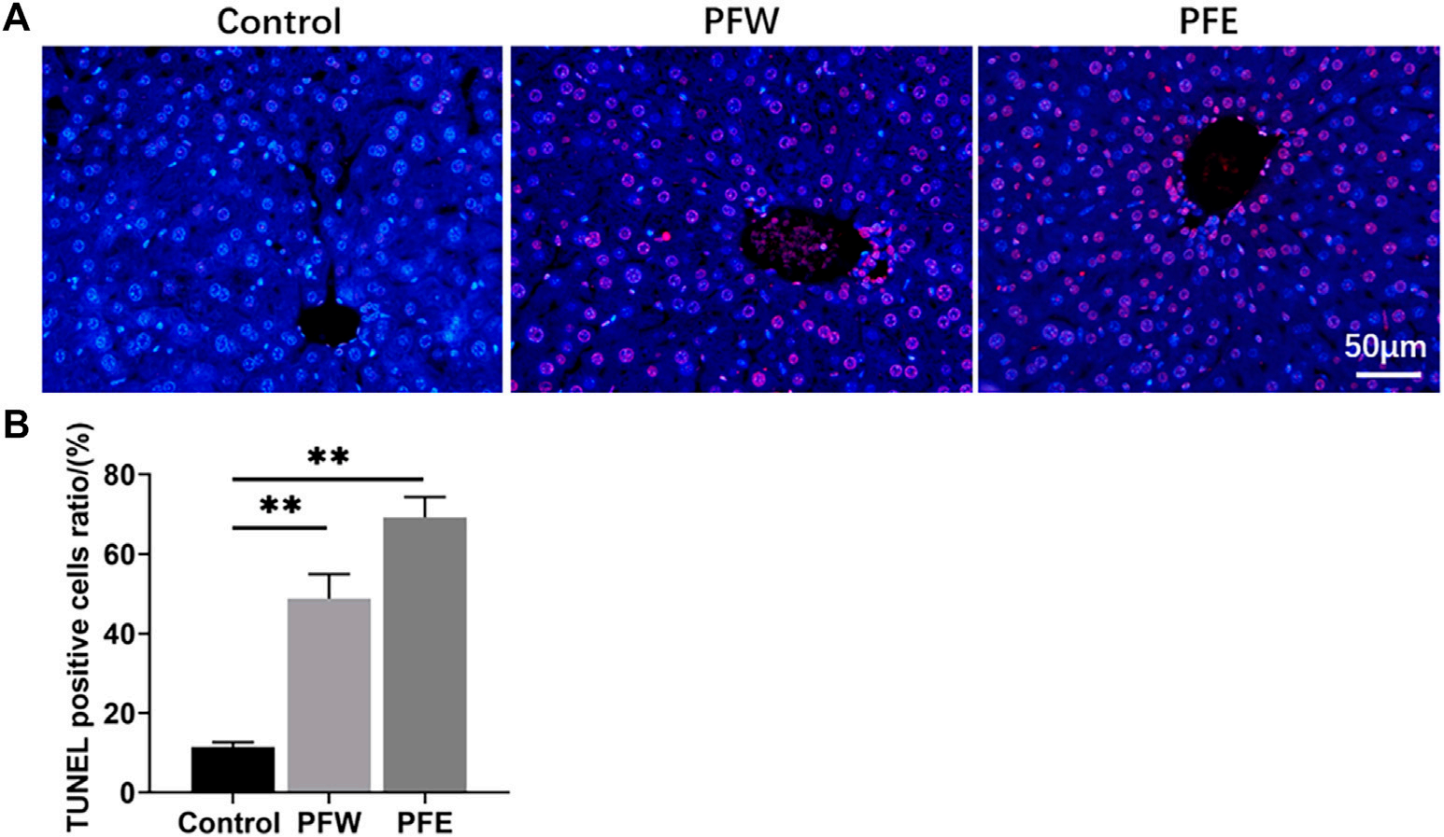
Detection of apoptotic hepatocytes in hepatic tissues using TUNEL assay. **(A)** Representative images of each group at ×400 magnification. **(B)** TUNEL-positive cell ratios in each group. Values were presented as mean ± SD and *n* = 4. ^*^ *p* < 0.05, ^**^ *p* < 0.01 versus the control group.

The morphological feature of liver tissues is direct and critical evidence for the diagnosis of liver damage (Shrestha et al., 2018). As shown in Figure 1A, 90% of mice in the PFE group demonstrated vacuolation. Besides, all mice in the PFE group showed varying degrees of hepatocellular steatosis, 30% of mice showed moderate lesion hepatocellular steatosis, and 90% of mice had hepatocellular hypertrophy (Table 1). However, only 20% of mice in the PFW group exhibited slight hepatocellular hypertrophy. PFE group mice showed significantly increased liver weight, liver/body weight ratio, and liver/brain weight ratio (*p* < 0.01) (Figures 1G–I). The direct bilirubin (DBIL) and TBIL in the rats of the PFE group were 1.67 and 1.37 times higher than those in the control (*p* < 0.05, *p* < 0.01) (Figures 1D,E). The ALT, AST, and indirect bilirubin (IBIL) in the rats of the PFE group were also higher than those in the control without significant difference (*p* = 0.07, 0.60, 0.07). Of note, the PFW treatment had little effect on hepatocyte phenotype. We used the TUNEL staining technique to analyze the effects of PF on apoptosis and investigate the potential PF-induced hepatotoxicity mechanisms. Here, the number of TUNEL-positive cells in the liver was considerably increased after PFW and PFE administration (Figure 2). Additionally, the PFE group had increased apoptosis cell number. Therefore, PFE caused more severe hepatotoxicity than PFW.

**TABLE 1:** Pathological manifestations of PFW and PFE in mice.

Pathological manifestations	Grading of lesion degree	Control	PFW	PFE
Hepatocellular steatosis	+	0	0	2
	2+	0	0	5
	3+	0	0	3
Hepatocellular hypertrophy	+	0	2	0
	2+	0	0	6
	3+	0	0	3

Note. “+” means a very slight lesion; “2+” means a mild lesion; and “3+” means a moderate lesion.

### Comparing the Composition Difference Between Water and Ethanol extracts of Psoraleae Fructus

**FIGURE 3:**
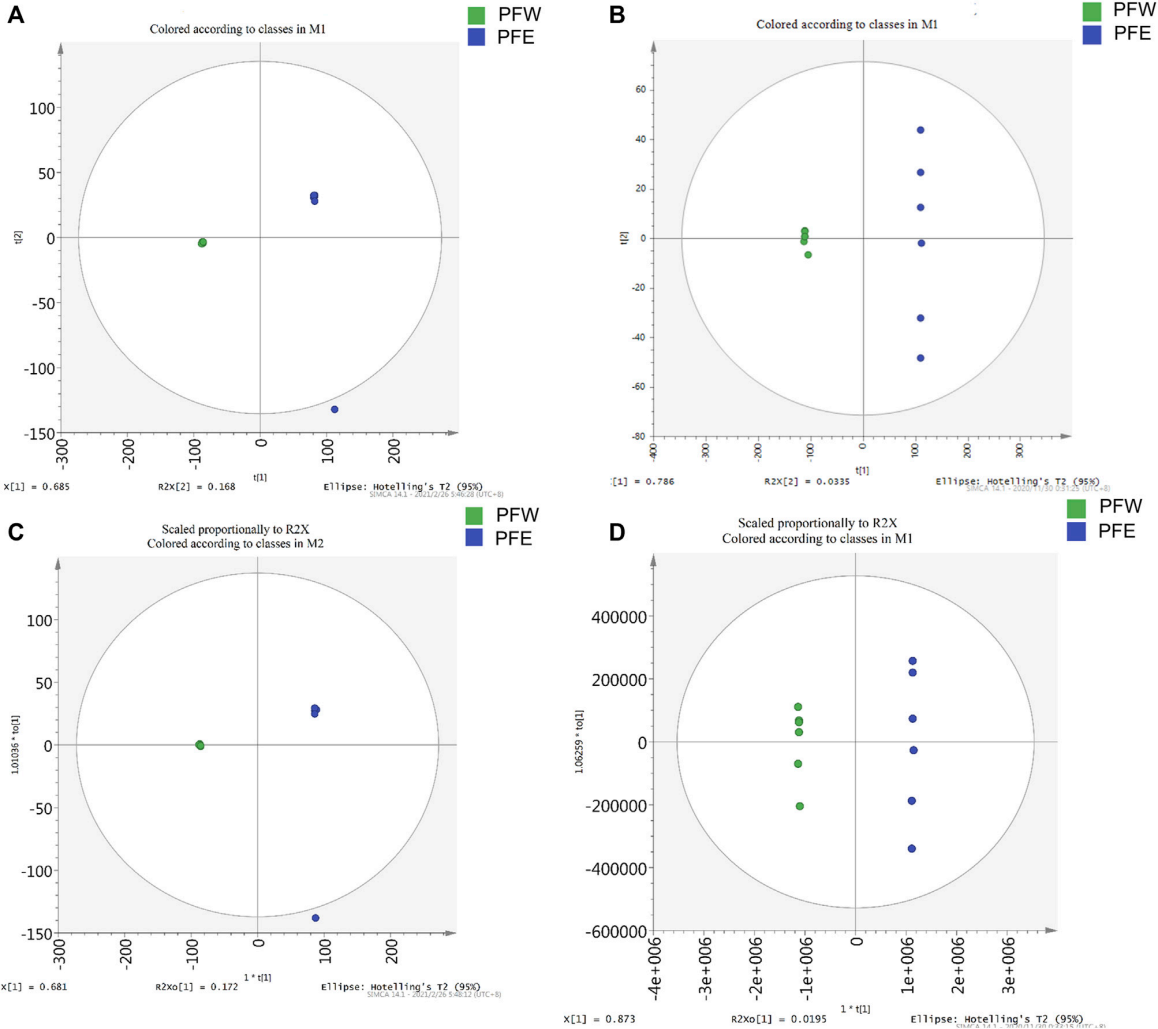
The PCA score plots and OPLS-DA models for the UHPLC-Q-Exactive MS analysis of PFW and PFE. **(A)** Negative ions mode of the PCA model. **(B)** Positive ions mode of the PCA model. **(C)** Negative ions mode of the OPLS-DA model. **(D)** Positive ions mode of the OPLS-DA model.

**FIGURE 4:**
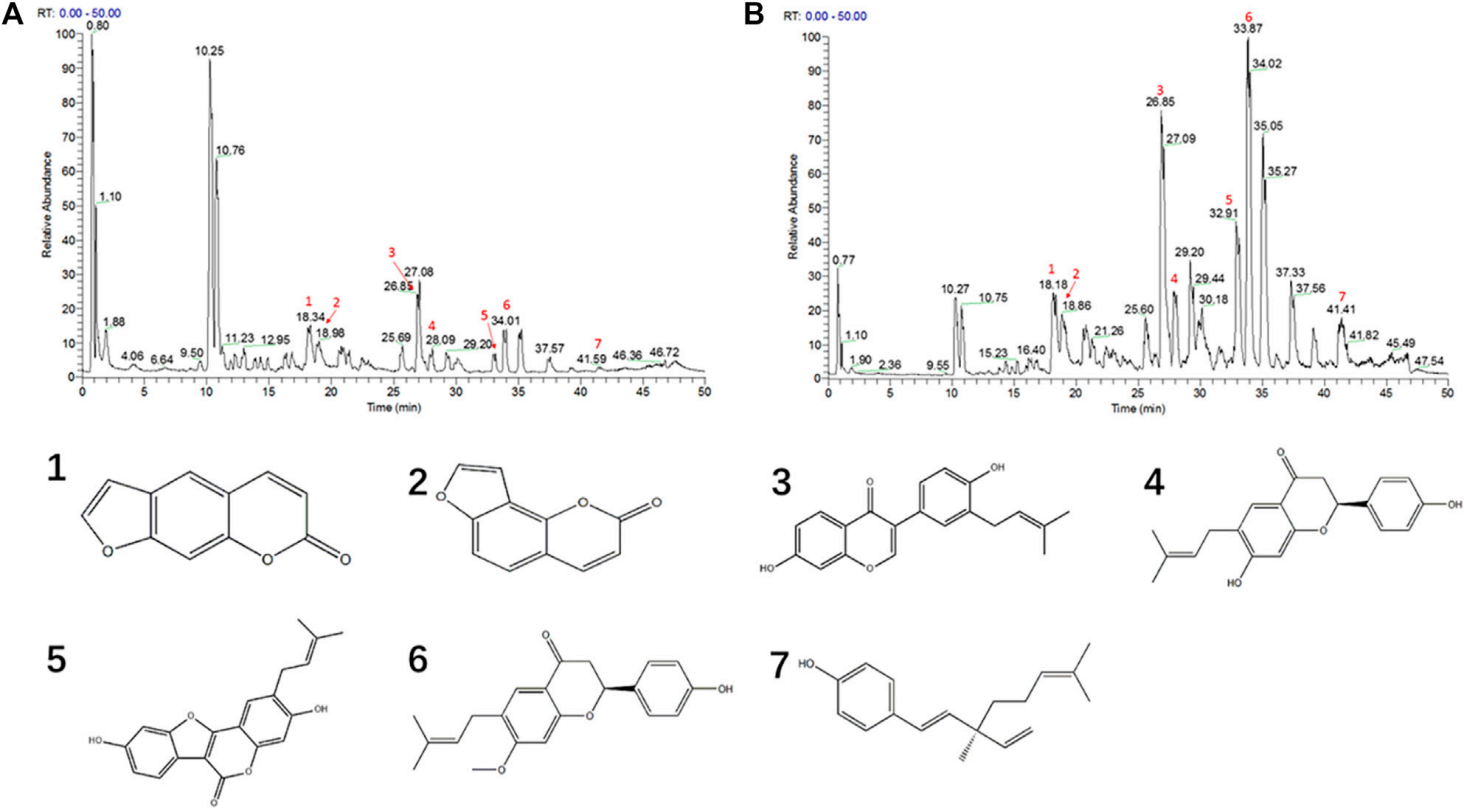
The UHPLC-Q-Exactive MS chromatogram of PC granules. **(A)** The PFW positive ion mode diagram. **(B)** The PFE positive ion mode diagram. **(C)** Different compound structures. 1, psoralen; 2, isopsoralen; 3, neobavaisoflavone; 4, bavachin; 5, psoralidin; 6, bavachinin; 7, bakuchiol.

Since PFE led to more severe hepatotoxicity than PFW, we speculated that the compounds enriched with PFE, rather than PFW, could be toxic constituents of PF. Therefore, we established a UHPLC-Q-Exactive MS platform to investigate the different PFE and PFW ingredient contents. Initially, unsupervised principal component analysis (PCA) illustrated that the trend in the PFW group was distinct from that in the PFE group (Figures 3A,B). This indicated that different extraction methods caused significant changes in the PF compositions. Then, the data were imported into the SIMCA-P version 13.0 software for multivariate analysis. All the samples were within the 95% CI (Hotelling’s T2 ellipse) with good separation, indicating that different extraction processes caused changes in the PF compounds. Orthogonal projections to latent structures discriminant analysis (OPLS-DA) model discrimination method was used to analyze the internal components in each group (Figures 3C,D). The card value screening was conducted according to the variable differentiation in the projection (VIP). The seven compounds were identified between PFW and PFE (VIP > 1 and *p* < 0.05). In PFE, the contents of psoralen, isopsoralen, bavachin, psoralidin, bavachinin, neobavaisoflavone, and bakuchiol were 1.64, 1.96, 9.34, 6.19, 8.21, 5.15, and 35.08 times higher than those in PFW, respectively (Figures 4A,B). As previously described, the main PF constituents were coumarins, flavonoids, and meroterpenes (Zhang et al., 2016). Thus, during our analysis, we chose the representative compounds of those chemical structure types. Remarkably, the above components with significant content differences included coumarins (psoralen, isopsoralen, and psoralidin), flavonoids (bavachin, bavachin, and neobavaisoflavone), and phenols (bakuchiol). Therefore, we speculated that these seven compounds could be potential PF toxic components.

### Effect of Potential Toxic Compounds on Cell Viability

**FIGURE 5:**
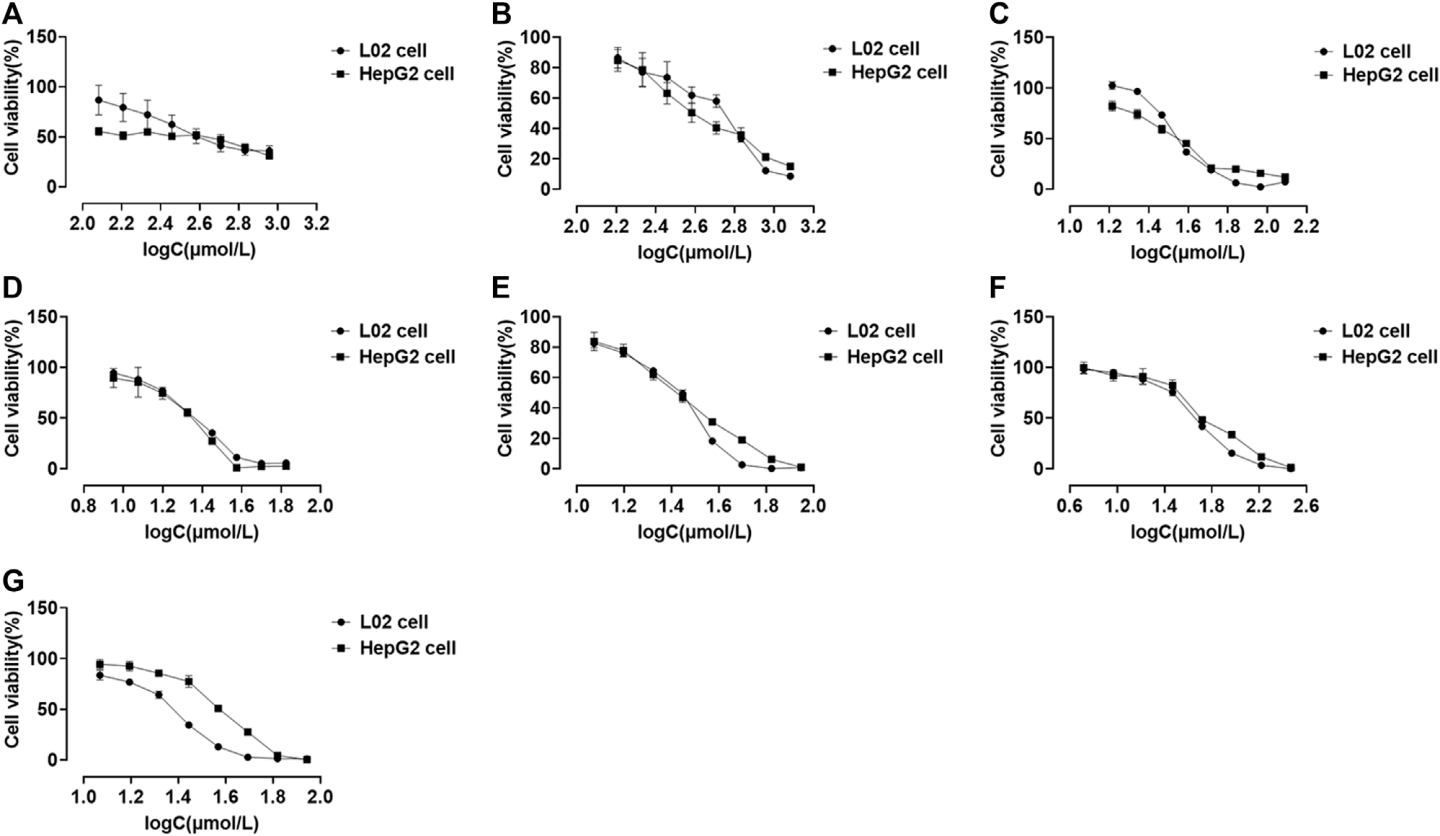
Cell viability of L02 and HepG2 cells treated with psoralen **(A)**, isopsoralen **(B)**, bavachin **(C)**, psoralidin **(D)**, bavachinin **(E)**, neobavaisoflavone **(F)**, and bakuchiol **(G)**. Values are presented as mean ± SD, *n* = 6.

We performed CCK-8 assays after 48 h of drug treatment to investigate the cytotoxic effects of compounds on the L02 and HepG2 cell lines. Eight series of concentrations were set for each compound. All compounds existed in a dose-dependent inhibitory manner (Figure 5). Psoralidin and bakuchiol (Figures 5D,G; Table 2) showed more potent inhibitory effects on cell viability than other compounds. The IC_50_ values for the L02 cell were 23.10 and 25.86 μmol/L, whereas those of the HepG2 cell were 23.36 and 34.49 μmol/L. Also, bavachin, bavachinin, and neobavaisoflavone showed intense repression of cell viability (Figures 5C,E,F). However, psoralen (Figure 5A) and isopsoralen (Figure 5B) showed no effect on HepG2 cell viability until they reached a concentration of 738.80 and 174.00 μmol/L. We, therefore, concluded that psoralen and isopsoralen had little hepatocyte toxicity. In contrast, bavachin, bavachinin, psoralidin, neobavaisoflavone, and bakuchiol could be potentially toxic compounds and could be used for further research.

**TABLE 2:** The IC_50_ values of all compounds.

Compounds	IC_50_ toward L02 cells (μmol/L)	IC_50_ toward HepG2 cells (μmol/L)
Psoralen	283.60	738.80
Isopsoralen	77.91	174.00
Bavachin	33.91	34.84
Psoralidin	23.10	23.36
Bavachinin	29.83	29.14
Neobavaisoflavone	47.78	51.69
Bakuchiol	25.86	34.49

### Effects of the Potential Toxic Compounds on the Release of Alkaline Phosphatase, Alanine Aminotransferase, and Aspartate Aminotransferase

**FIGURE 6:**
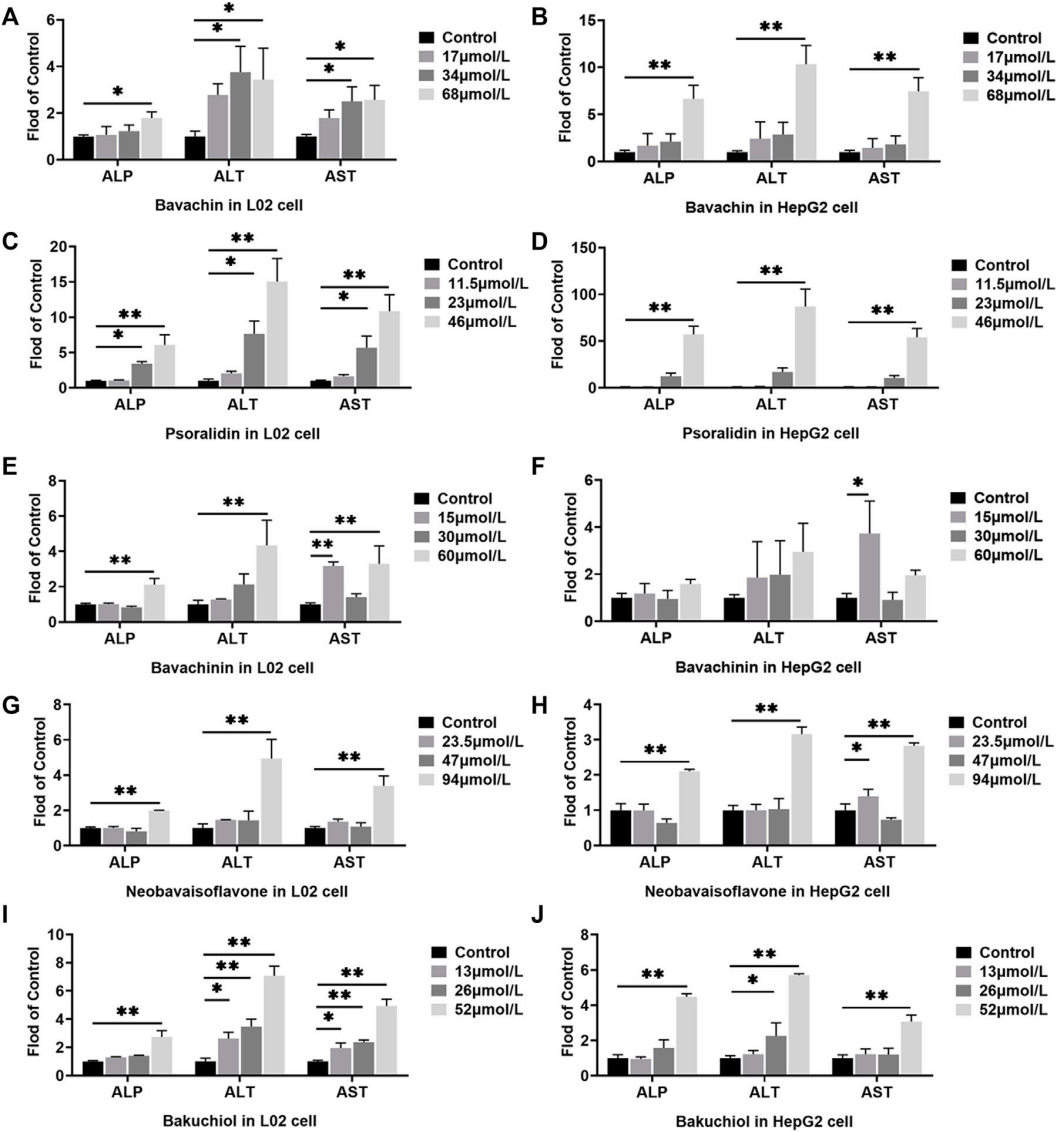
Effects of different doses of bavachin **(A,B)**, psoralidin **(C,D)**, bavachinin **(E,F)**, neobavaisoflavone **(G,H)**, and bakuchiol **(I,J)** on the ALP, ALT, and AST levels in L02 and HepG2 cells. The values are presented as mean ± SD and *n* = 6. ^*^ *p* < 0.05, ^**^ *p* < 0.01 versus the control group. ALP, alkaline phosphatase; ALT, alanine aminotransferase; AST, aspartate aminotransferase.

ALP, ALT, and AST levels in cell culture supernatant were measured to evaluate the cell damage of the potentially toxic compounds. Here, we established that all the five compounds increased the ALP, ALT, and AST levels in both L02 and HepG2 cells (*p* < 0.05, *p* < 0.01, Figure 6). In the HepG2 cells treated with psoralidin (46 μmol/L), ALP, ALT, and AST levels were 57, 87, and 54 times higher than those cells in the control group, respectively (Figure 6D). On the contrary, bakuchiol increased ALP and AST levels in the L02 cells at all dose concentrations (Figure 6I). Finally, bavachin, bavachinin, and neobavaisoflavone increased ALP, ALT, and AST contents at high dosage in L02 cells (Figures 6A,B,E–H). These results suggested that bakuchiol and psoralidin caused obvious cytotoxicity.

### Effect of Potential Toxic Compounds on Apoptosis

**FIGURE 7:**
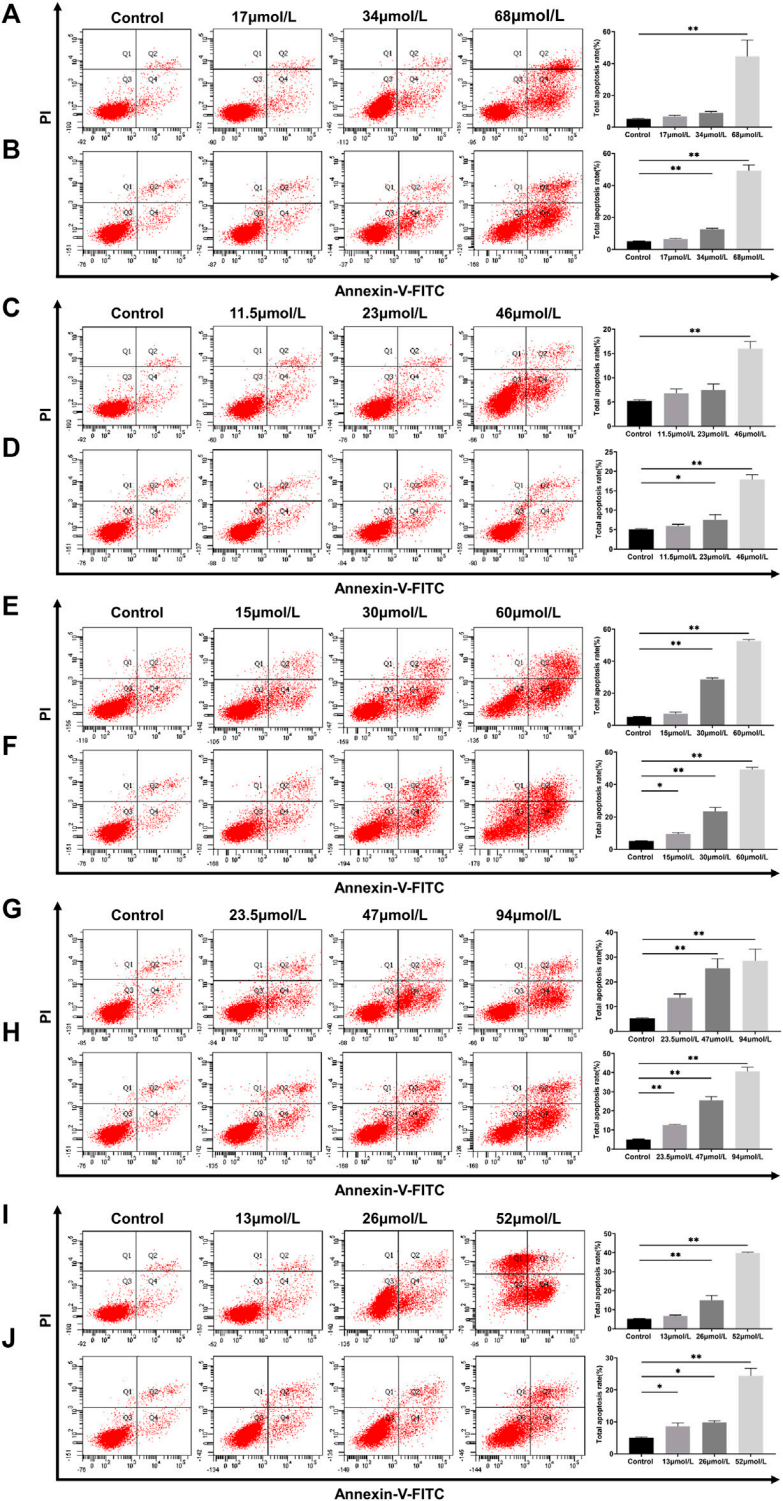
Apoptosis of L02 and HepG2 cells treated with bavachin **(A,B)**, psoralidin **(C,D)**, bavachinin **(E,F)**, neobavaisoflavone **(G,H)**, and bakuchiol **(I,J)**. These values are presented as mean ± SD and *n* = 3. ^*^ *p* < 0.05, ^**^ *p* < 0.01 versus the control group.

Apoptosis is a common physiological mechanism that eliminates cells in DILI (Jacobson et al., 1997). Flow cytometry assays with the Annexin V-FITC/PI double-staining technique were performed to investigate the effect of potential toxic compounds on L02 and HepG2 cells apoptosis. After the L02 cells were incubated with bavachin for 48 h, the apoptotic cell percentage increased from 6.7% to 44.47% (Figure 7A). A similar result was found in HepG2 cells (Figure 7B).

Interestingly, psoralidin had little effect on cell apoptosis, as it only increased the apoptotic cell percentage to 17% at 46 μmol/L (Figure 7C,D). On the other hand, neobavaisoflavone (Figures 7G,H) and bakuchiol (Figures 7I,J) treatments induced apoptosis in a dose-dependent manner. Also, bakuchiol treatment-induced necrocytosis increased the necrotic cell percentages to 34.6% at 52 μmol/L (Figure 7I). More importantly, bavachinin (Figures 7E,F) and bakuchiol (Figures 7I,J) induced cell apoptosis in low dose concentrations of 15 and 13 μmol/L, respectively. These data were similar to those obtained in the TUNEL staining *in vivo* setup.

### Effects of the Potential Toxic Compounds on the Intracellular Lipid

**FIGURE 8:**
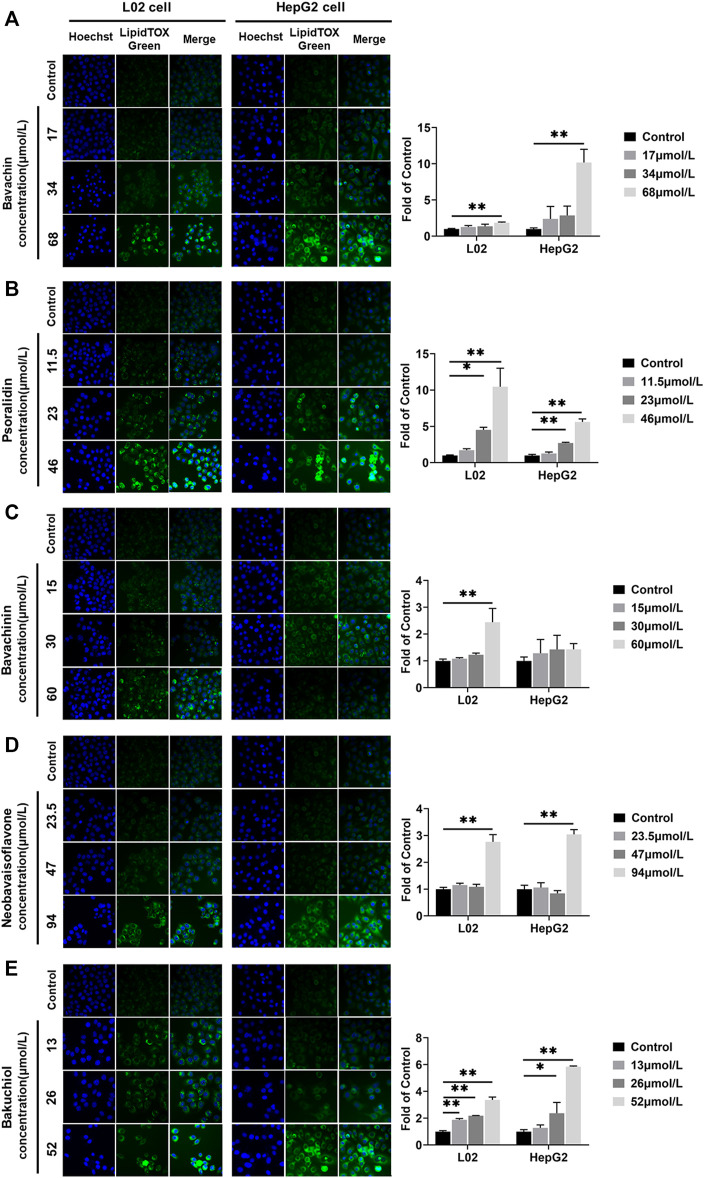
Lipids were examined and quantified by HCS LipidTOX Green staining assays after exposure of L02 and HepG2 cells to the five compounds. **(A)** Bavachin; **(B)** psoralidin; **(C)** bavachinin; **(D)** neobavaisoflavone; **(E)** bakuchiol. Values are presented as mean ± SD, *n* = 3. ^*^ *p* < 0.05, ^**^ *p* < 0.01 versus the control group. HCS, high-content screening.

All compounds increased the L02 and HepG2 cells lipid accumulation levels (*p* < 0.05, *p* < 0.01, Figure 8). The lipid accumulations in L02 cells treated with bakuchiol at 13, 26, and 52 μmol/L were 1.88, 2.18, and 3.37 times higher than in the control group, respectively. The lipid accumulations in HepG2 cells increased by bakuchiol treatment were 1.27, 2.37, and 5.84 times higher than in control (Figure 8E). Lipid accumulation was increased after psoralidin treatment at 23 and 46 μmol/L (Figure 8B). Besides, bavachin, bavachinin, and neobavaisoflavone promoted lipid accumulation in L02 and HepG2 cells (*p* < 0.05, *p* < 0.01, Figures 8A,C,D). This demonstrated that the balance between lipid synthesis and metabolism in hepatocytes was disrupted in these compounds-treated group. Notably, this finding was consistent with our previous *in vivo* study (Guo et al., 2020).

### Effects of the Potential Toxic Compounds on Generating ROS

**FIGURE 9:**
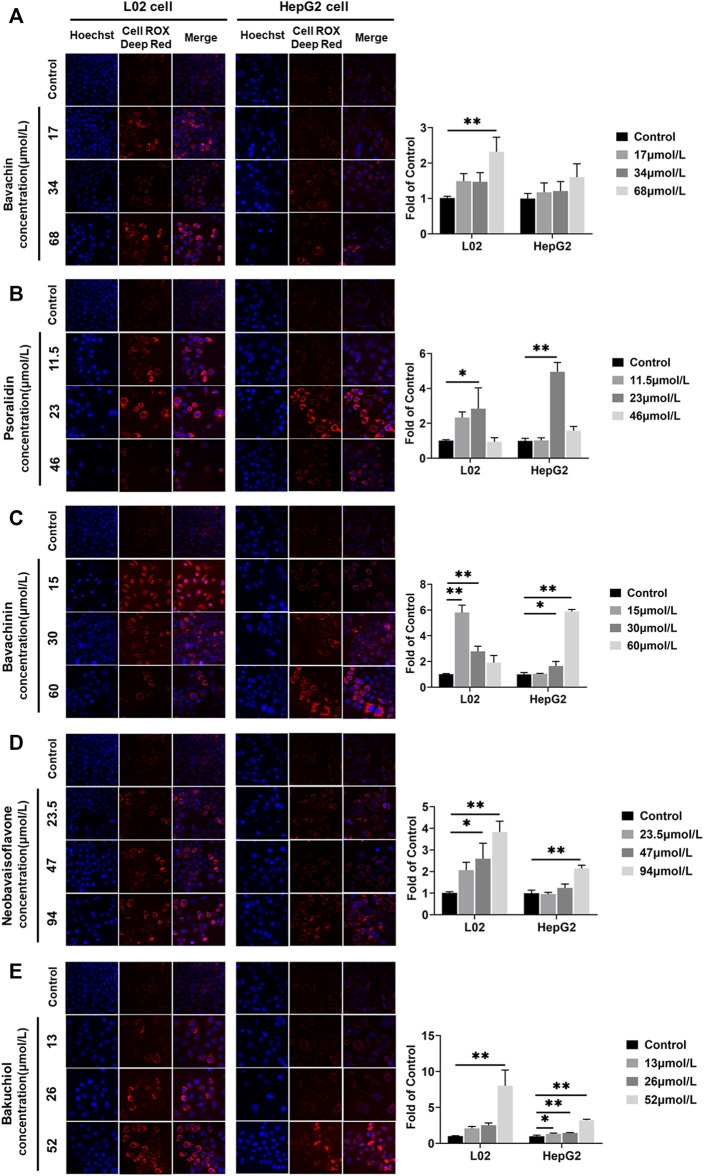
ROS was examined and quantified using the CellROX Deep Red Reagent staining assays after the five compounds were exposed in L02 and HepG2 cells. **(A)** Bavachin; **(B)** psoralidin; **(C)** bavachinin; **(D)** neobavaisoflavone; **(E)** bakuchiol. Values are presented as mean ± SD, *n* = 3. ^*^ *p* < 0.05, ^**^ *p* < 0.01 versus the control group. ROS, reactive oxygen species.

ROS levels in L02 and HepG2 cells were increased after the five compounds incubation for 24 h (Figure 9) (*p* < 0.05,*p* < 0.01). Psoralidin induced ROS generation at 23 μmol/L (Figure 9B), bavachinin increased the ROS level at 30 and 60 μmol/L in HepG2 cells (Figure 9C), whereas bavachin only increased the ROS level at 68 μmol/L in L02 cells, and neobavaisoflavone increased the ROS level at 47 and 94 μmol/L (Figures 9A,D). Bakuchiol promoted ROS generation at all doses in HepG2 cells (Figure 9E), suggesting that it is more toxic on hepatocytes than the other compounds.

### Effects of the Potential Toxic Compounds on MMP

**FIGURE 10:**
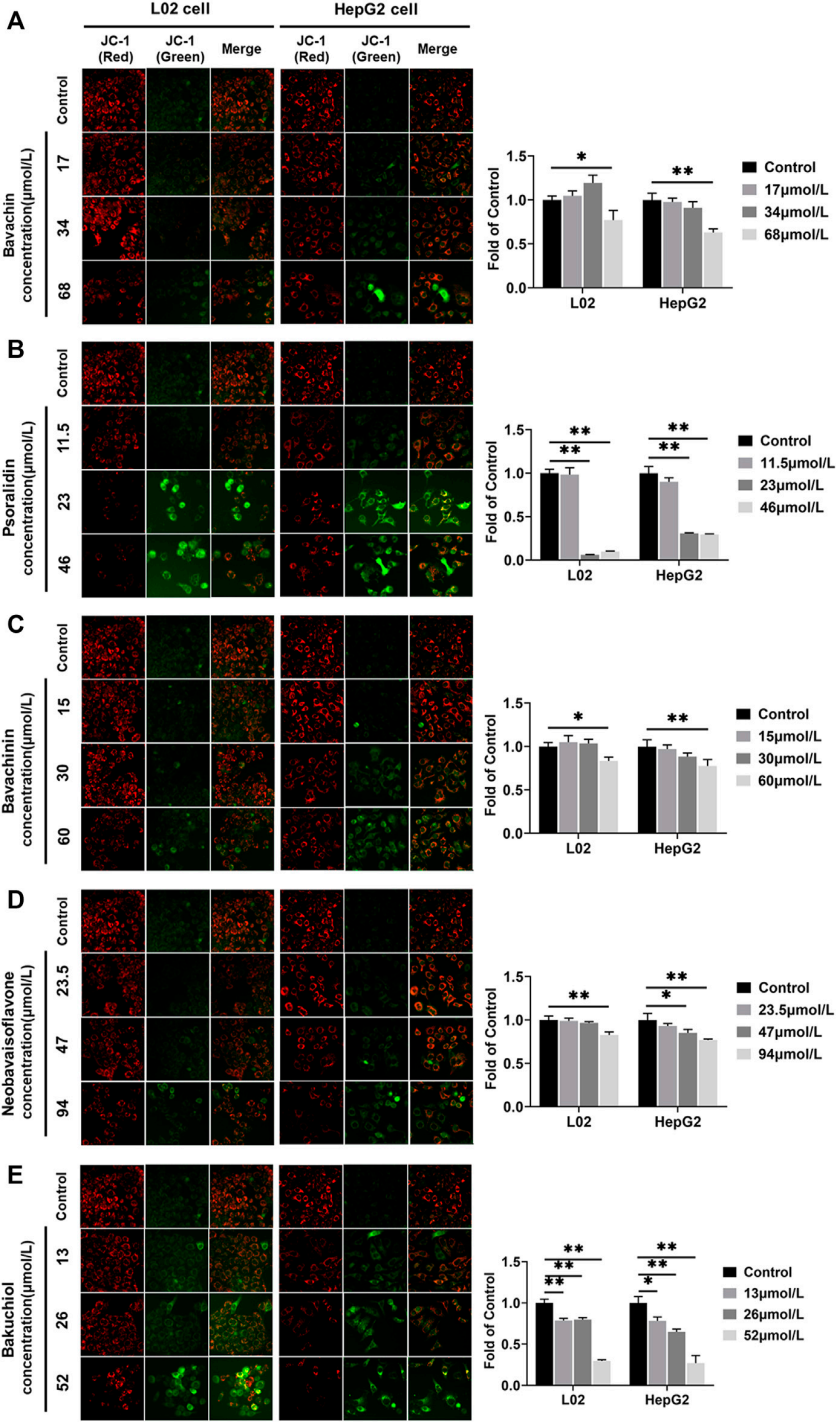
MMP was examined and quantified using JC-1 staining assays after five compounds were exposed in L02 and HepG2 cells. **(A)** Bavachin; **(B)** psoralidin; **(C)** bavachinin; **(D)** neobavaisoflavone; **(E)** bakuchiol. All values are presented as mean ± SD and n = 3. ^*^ *p* < 0.05 and ^**^ *p* < 0.01 versus the control group. MMP, mitochondrial membrane potential.

MMP was directly used to clarify whether these five compounds influenced mitochondrial function. We found that the MMP fluorescence intensities were significantly lower in compound-treated groups than in control (*p* < 0.05, *p* < 0.01) (Figure 10). Furthermore, psoralidin significantly reduced the MMP at 23 and 46 μmol/L concentrations (Figure 10B), and bakuchiol significantly reduced the MMP of L02 and HepG2 cells at 13, 26, and 52 μmol/L concentrations, indicating mitochondrial damage (Figure 10E). Besides, bavachin, bavachinin, and neobavaisoflavone reduced the MMP levels at 68, 60, and 94 μmol/L concentrations, respectively (Figures 10A,C,D). This result suggested that psoralidin and bakuchiol caused significant damage to mitochondria at low doses.

## Discussion

This study conducted a comparative experiment on the PF hepatotoxic components and preliminarily explored its mechanism using *in vitro* models. First, we have evaluated the PFW and PFE hepatotoxicity levels and revealed that the hepatotoxicity of PFE was more evident than PFW. Next, we have compared and analyzed the PFW and PFE chemical constituents using the UHPLC-Q-Exactive MS method and identified 50 components of PF (data was not shown), and we found that the psoralen, isopsoralen, bavachin, psoralidin, bavachinin, neobavaisoflavone, and bakuchiol contents were significantly different in these two extracts. Moreover, *in vitro* experimental setups revealed that all components showed a dose-dependent inhibition proliferation manner in each cell type, except psoralen and isopsoralen. Unlike other previous researches, this study demonstrated that psoralen and isopsoralen had little hepatocyte toxicity. Bavachin, psoralidin, bavachinin, neobavaisoflavone, and bakuchiol induced hepatocyte injury, including cell apoptosis and lipid accumulation, via mitochondrial damage caused by an abnormally increased ROS. Thus, our study confirmed that PF had hepatotoxicity. Also, bavachin, psoralidin, bavachinin, neobavaisoflavone, and bakuchiol were the main toxic compositions, and among them, psoralidin and bakuchiol were more hepatotoxic. This confirmed the underlying mechanism related to ROS accumulation and mitochondrial damage.

Recently, research on HILI has increased with the extensive worldwide application of CM and HM. PF was first recorded in Lei Gong’s Treatise on Preparation and Broiling of Materia Medica (Leigong Paozhi Lun). However, there have been increasing studies on liver injury associated with PF in recent years (Zhao et al., 2016; Zhou et al., 2017; Li and Cheng, 2018; Wang and He, 2018; Wu and Yao, 2018; Li A. et al., 2019; Liu and Wu, 2019). Numerous studies have shown that raw powder, aqueous PF extracts, and alcoholic PF extracts exhibited hepatotoxicity in rats (Xu et al., 2017; Wang Y. et al., 2019; Yang et al., 2019; Liu et al., 2021). Our previous experiments had indicated that the toxicity of the ethanol extract was more substantial than that of the water extract in rats and mice (Guo et al., 2020). This suggested that ethanol extraction technology could enrich toxic constituents. Therefore, water extraction technology is recommended during routine clinical application. Furthermore, some post-marketing TCM, like Xian Niu Jian Gu granules (Wang and Dong, 2008), in which the PF was extracted using ethanol, displayed evident hepatotoxicity. Notably, hundreds of compounds belonging to various groups have been separated from PF, with coumarins, flavonoids, and meroterpenes being more dominant (Zhang et al., 2016). Elsewhere, studies have reported that the ethanol extraction process significantly increased the psoralen, isopsoralen (Di, 2019), and bakuchiol contents (Li J. M. et al., 2017). This study had analyzed and compared the compounds between PFW and PFE extracts using the UHPLC-Q-Exactive MS method. Our findings were consistent with previously reported studies (Li J. M. et al., 2017; Di, 2019). Here, we found that ethanol extract technology-enriched coumarins (psoralen, isopsoralen, and psoralidin), flavonoids (bavachin, bavachinin, and neobavaisoflavone), and monoterpenes (bakuchiol), suggesting that the PF hepatotoxic ingredients were related to these compounds.

CCK-8 assay revealed that the IC_50_ values of psoralidin and bakuchiol were the lowest, nearly 25 μmol/L, suggesting maximum toxicity. Bavachin and bavachinin were the second most toxic compounds, as the IC_50_ value was approximately 30 μmol/L, and were followed by neobavaisoflavone. Psoralen and isopsoralen had very low or no toxicity for hepatic cell viability. This result was consistent with another report, showing that psoralen inhibited 50% cell viability at almost 400 μmol/L (Yu et al., 2020). ALT and AST mainly exist in liver cells and are translocated to the outer membrane from the interior once necrosis or liver damage has occurred (Ramaiah, 2007). They are sensitive indicators for examining liver function in clinical treatment and assessing the damage caused to liver cells. Our study had demonstrated that psoralidin and bakuchiol increased the ALP, ALT, and AST levels by five times, followed by bavachin, bavachinin, and neobavaisoflavone. These results had been proved using the CCK-8 assay.

These five compounds had potentially inhibited L02 and HepG2 cell proliferation via the apoptosis-associated processes. Apoptosis, a highly structured and ordered process, eliminates superfluous, harmful, and metabolically perturbed cells and is a basic form of cell death (Jacobson et al., 1997). Our study has demonstrated that apoptosis was induced in L02 and HepG2 cells by bavachin, psoralidin, bavachinin, neobavaisoflavone, and bakuchiol. This has been confirmed using a flow cytometry assay containing Annexin V/PI double-staining technique. Compared with the control, bavachin, bavachinin, neobavaisoflavone, and bakuchiol had markedly and progressively increased the cell early apoptosis percentage as the concentration increased. Based on these data and established pharmacological research (Li F. et al., 2017; Huang et al., 2019; Yuan et al., 2019), we supposed that monoterpenes and flavonoids could be toxic constituents, whereas coumarins would be active ingredients. Thus, the over-enrichment of these two types of components should be avoided in clinical applications to ensure medication safety.

In many compounds, oxidative stress plays a significant role in promoting toxicity mechanisms, whether by producing free radicals or depleting cellular antioxidant capacity (Shi et al., 2018). This study had found that the ROS levels in L02 and HepG2 cells were increased after being treated with five compounds. Meanwhile, psoralidin and bakuchiol have shown a strong induction of ROS content. On the one hand, increased ROS could injure proteins, DNA, and lipids, by promoting cell death through activating stress-related signaling pathways (Luedde et al., 2014). Similar findings have been illustrated in HepaRG cells treated with bavachinin, resulting in ROS generation and oxidative damage (Wang S. et al., 2018). Additionally, excess ROS could induce lipid peroxidation (Donato and Tolosa, 2021). Elsewhere, normal hepatocyte lipid metabolism has been shown as the balance between fatty acid synthesis (lipogenesis) and fat catabolism via β-oxidation (lipolysis) (Wu et al., 2017). Nevertheless, high ROS production could disrupt this balance and cause lipid accumulation (Donato and Tolosa, 2021). The liver is the main organ of fat metabolism in the body; the accumulation of lipid droplets could trigger liver steatosis injury (O’Brien et al., 2006). In our study, PF administration led to hepatocyte steatosis, as demonstrated using *in vivo* setups. Moreover, bavachin, psoralidin, and bakuchiol significantly promoted the intracellular lipid deposition of L02 and HepG2 cells. Hence, we supposed that PF and its main toxic compounds induced hepatocyte steatosis through ROS accumulation.

Mitochondria serve as the primary source of ROS in mammals (Li et al., 2020). During injury, the ROS production rates were increased in the mitochondria or other cell compartments (Pereira et al., 2012). Our experiments revealed that all five compounds (bavachin, psoralidin, bavachinin, neobavaisoflavone, and bakuchiol) reduced the MMP levels. Furthermore, high psoralidin and bakuchiol doses had shown the most significant effect on the MMP repression, and this was consistent with the induction effect of psoralidin and bakuchiol on ROS. Also, ROS could influence the functioning of the mitochondria. ROS production and removal imbalance had resulted in cumulative ROS that contacts mitochondria and cellular components, leading to mitochondrial oxidative damage (Dan Dunn et al., 2015). The mitochondrial permeability transition pore (mPTP) located in the mitochondrial inner membrane could remain open to increase ROS levels. Mitochondrial permeability transition could induce mitochondrial depolarization and swelling, decrease electron transport chain (ETC) activity, and release apoptotic factors (Chen et al., 2015; Ye et al., 2016). Besides, mtDNA lacking histone protection is highly sensitive to ROS and prone to damage and mutations under oxidative stress, resulting in respiratory chain defects and decreased mitochondrial biogenesis (Bouchez and Devin, 2019; Kaarniranta et al., 2019). Based on these studies, we have speculated that bavachin, psoralidin, bavachinin, neobavaisoflavone, and bakuchiol could induce mitochondrial damage and increase ROS production. In return, the increased ROS disrupted mitochondrial function.

Usually, the drug-induced hepatocyte injury mechanism is divided into three stages: initial hepatocyte injury (cell stress and mitochondrial inhibition), mitochondrial permeability transition, and hepatocyte death (apoptosis and necrosis) (Russmann et al., 2009; Suh, 2020). Similarly, in this study, the mechanism of PF-induced hepatotoxicity had also been categorized into the following three stages: ROS accumulation, MMP repression, and hepatocyte apoptosis and steatosis. Moreover, bavachin, psoralidin, bavachinin, neobavaisoflavone, and bakuchiol could be the primary toxic constituents. Among them, psoralidin and bakuchiol shown more toxicity than other compounds. Nonetheless, this study was limited since it lacks proper mechanisms to show how MMP is reduced in the five compounds using *in vivo* experiments setups.

Most studies on PF induced hepatotoxicity have focused on the toxicity and mechanism of single compound in PF, including bakuchicin, psoralen, isopsoralen, bakuchiol, and bavachinin (Kim et al., 2016; Li ZJ. et al., 2017; Wang S. et al., 2018; Yang et al., 2018; Yu et al., 2019). However, TCM has multi-component, multi-pathway, and multi-target characteristics. The single-component study ignores the integrity of TCM. So a systematic analysis of the toxic constituent and mechanism was needed. This study first compared the hepatotoxicity of PFW and PFE and discovered that PFE induced more severe liver injury than PFW. We found that the contents of psoralen, isopsoralen, bavachin, psoralidin, bavachinin, neobavaisoflavone, and bakuchiol in PFE were much higher than those in PFW. Subsequently, we had clarified the hepatotoxicity level of these compounds using the CCK-8 assay and high-content screening analysis. The results showed that bavachin, psoralidin, bavachin, neobavaisoflavone, and bakuchiol inhibited cell proliferation, but psoralen and isopsoralen had little inhibition effect on cell viability. Interestingly, psoralidin exhibited a strong induction effect on ALP, ALT, and AST contents (more than fivefold when the dose concentration of psoralidin was 23 mol/L). However, it had a minimal impact on apoptosis (only 15% of the cells showed apoptosis at a high dose concentration). On the other hand, bakuchiol had exhibited a strong induction effect on lipid accumulation (bakuchiol-induced lipid accumulation at low dose) but had little effect on ALP, ALT, and AST contents. We generally believed that these compounds had a different impact on hepatocytes and collectively led to hepatotoxicity in all five compounds rather than a single compound, which was the main finding of this study.

This research has tried a novel approach to screen the toxic PF constituents. PFE had induced more severe hepatotoxicity than PFW since the PFE compounds had high toxic constituent contents than PFW. The classic discovery method of toxic compounds from TCM based on chemical separation-structure identification-activity detection had a large workload and tedious operation. Recently, serum pharmacochemistry (Yang et al., 2020), network pharmacology (Wang N. et al., 2019), spectrum–effect relationship (Li W. et al., 2019), high-throughput screening (Ma et al., 2016; Li and Xia, 2019), and metabonomics (Wu et al., 2018) were developed to solve the problem. However, there was still no perfect way to discover the scientific meanings of the toxic herb. Different from existing herbal toxic constituent screening methods, this study was based on the *in vivo* experimental data and established a novel research approach by comparing PFW (low toxic) and PFE (high toxic) and finding constituent differences. We had simultaneously compared and analyzed multiple compounds, which were more consistent with the manifestations of PF-induced hepatotoxicity in clinical practice. This was the second finding of this study.

HepG2 is an immortalized human hepatoma cell line, and L02 is an immortalized hepatocyte cell line (Chen et al., 2020). L02 and HepG2 cells were commonly used in toxicity studies. But there were some differences between these two cell lines. Recent research found that some drugs, especially anticancer drugs, exhibited high cell selectivity between HepG2 cells and L02 cells, which express high and low ανβ3 integrin, respectively (Mugaka et al., 2021). And HepG2 cells and L02 cells responded to the same stimuli with various reactions. Gao had found that garlic flavonoids alleviate H_2_O_2_-induced oxidative damage in L02 cells but induce apoptosis in HepG2 cells (Gao et al., 2021). Other research revealed that CYC1 and HPRT1 were considered as the most stable reference genes in HepG2 cells and that TUBB2a was the steadiest one in L02 cells when treated with ethanol (EtOH), hydrogen peroxide (H_2_O_2_), acetaminophen (APAP), and carbon tetrachloride (CCl_4_) (Chen et al., 2020). Frankly, we were not sure which cell line was more suitable for hepatotoxicity study. We supposed that HepG2 cells were more sensitive to toxic compounds than L02 cells and that L02 cells were more similar to normal human hepatocytes. Using these two cells made an evaluation of PF hepatotoxicity more convincing and credible. In our study, five compounds induced apoptosis more strongly in HepG2 cells than L02 cells. In particular, psoralidin (23 and 46 mol/L) and bavachinin (60 mol/L) showed a more substantial effect on lipid accumulation in L02 cells than HepG2 cells. In contrast, bavachin (68 mol/L) had a stronger inducing effect on lipid accumulation in the HepG2 cells. Additionally, the neobavaisoflavone (47 mol/L and 94 mol/L) and bakuchiol (52 mol/L) compounds had a stronger effect on ROS induction in L02 cells than HepG2 cells. We speculated that this was related to functional differences between hepatocytes. Nevertheless, the comprehensive analysis results of the two cell lines are equally representative. This also demonstrated that our findings are convincing and credible for evaluating PF liver toxicity. This was our third finding.

In conclusion, this study used L02 and HepG2 cells as *in vitro* models and conducted a comparative and analytical study of PFE (high toxicity) and PFW (low toxicity) to explore the hepatotoxic compounds of PF for the first time; bavachin, psoralidin, bavachinin, neobavaisoflavone, and bakuchiol were the main hepatotoxic components of PF, and psoralidin and bakuchiol were more hepatotoxic. The ethanol extraction process aggravated the hepatotoxicity of PF. PF is very commonly used in clinical practice, and the present study clarifies the hepatotoxic components and preliminary toxicity mechanism of PF, which has important clinical significance. Currently, DILI has become a significant cause of drug development failure and withdrawal. This study provides a reference for the drug evaluation of new drugs containing PF, and ethanol extraction of PF should be avoided in the drug extraction process study. Moreover, this study also provides a reference for the development of herbal standards for PF, which may limit the content of toxic components in future herbal standards.

This study had limitations, including a lack of a deeper understanding of the mechanism underlying how the five compounds induce mitochondrial damage and lipid accumulation. Besides, it has not verified hepatotoxicity *in vivo* models. In the future, we will carry out *in vivo* experiments to verify the PF hepatotoxic compounds and conduct an in-depth study on the mechanism underlying PF-induced liver injury.

## Conclusion

In summary, we conducted a comparative experiment on the PF hepatotoxic components and preliminarily explored its mechanism using *in vitro* models. We revealed that the hepatotoxicity of PFE was more evident than PFW. And we found that the psoralen, isopsoralen, bavachin, psoralidin, bavachinin, neobavaisoflavone, and bakuchiol contents were significantly different in PFW and PFE. Moreover, *in vitro* experimental setups revealed that all components showed a dose-dependent inhibition proliferation manner in each cell type, except psoralen and isopsoralen. Bavachin, psoralidin, bavachinin, neobavaisoflavone, and bakuchiol have induced hepatocyte injury, including cell apoptosis and lipid accumulation, via mitochondrial damage caused by an abnormally increased ROS. Thus, our study confirmed that PF had hepatotoxicity, and the mechanism could be related to oxidative stress and mitochondrial damage-mediated apoptosis.

## Data Availability

The original contributions presented in the study are included in the article/Supplementary Material. Further inquiries can be directed to the corresponding authors.
